# Cross-Cultural Validation of the Malaysian Mood Scale and Tests of Between-Group Mood Differences

**DOI:** 10.3390/ijerph20043348

**Published:** 2023-02-14

**Authors:** Philip Chun Foong Lew, Renée L. Parsons-Smith, Andrea Lamont-Mills, Peter C. Terry

**Affiliations:** 1 Sport Performance Division, National Sports Institute of Malaysia, Kuala Lumpur 57000, Malaysia; 2 Centre for Health Research, University of Southern Queensland, Toowoomba, QLD 4350, Australia; 3 School of Psychology and Wellbeing, University of Southern Queensland, Toowoomba, QLD 4350, Australia; 4 Pearson Online Learning Services, Pearson, Melbourne, VIC 3008, Australia; 5 Academic Affairs Division, Ipswich Campus, University of Southern Queensland, Ipswich, QLD 4305, Australia; 6 Graduate Research School, University of Southern Queensland, Toowoomba, QLD 4350, Australia

**Keywords:** BRUMS, MASMS, Malaysia, sport, athlete, mood, cross-cultural, translation, validation

## Abstract

Mood measures have been shown to have utility for monitoring risks to mental health and to predict performance among athletes. To facilitate use in a Malaysian context, we tested a Malay-language version of the 24-item Brunel Mood Scale (BRUMS), referred to as the Malaysian Mood Scale (MASMS). Following a thorough translation–back-translation process, the 24-item MASMS was administered to 4923 Malay-speaking respondents (2706 males, 2217 females; 2559 athletes, 2364 non-athletes), ranging in age from 17 to 75 years (*M* = 28.2 years, *SD* = 9.4 years). Confirmatory factor analysis supported the six-factor MASMS measurement model (CFI = 0.950, TLI = 0.940, RMSEA = 0.056 [CI 0.055, 0.058]). Convergent and divergent validity of the MASMS were supported via relationships with depression, anxiety, and stress measures. Significant differences in mood scores were found between athletes and non-athletes, males and females, and younger and older participants. Tables of normative data and profile sheets for specific groups were generated. We propose that the MASMS is a valid measure that can be used to monitor mental health status among athletes and non-athletes and that facilitates future mood-related research in Malaysia.

## 1. Introduction

There has been persistent interest in investigating mood as a construct in sport and exercise domains [1,2,3,4]. Mood has been defined [2] as “a set of feelings, ephemeral in nature, varying in intensity and duration, and usually involving more than one emotion” (p. 16). Historically, the most frequently used instrument to assess mood has been the Profile of Mood States (POMS; [5]), a self-report inventory of six mood dimensions: Tension, depression, anger, vigour, fatigue, and confusion. The POMS was initially used to assess mood in clinical populations and was then extended to college student populations [5]. It has subsequently been shown to be valid for use with athletes in sport and exercise settings and has been used in many sport-related studies [6].

The original 65-item POMS requires a relatively lengthy completion time of 8–10 min, which has resulted in numerous truncated versions being developed [7,8,9,10]. Terry and Lane developed and validated a 24-item short version, designed primarily for use in sport and exercise domains, now known as the Brunel Mood Scale (BRUMS) [11,12]. The 24-item, 6-factor BRUMS has undergone rigorous validity testing and has demonstrated satisfactory predictive, concurrent, criterion, and factorial validity, and appropriate test–retest reliability [11,12].

Mood profiling is a process in which mood scale scores are plotted against normative scores to provide a graphical representation of mood states [3]. Its application in sports gained popularity following studies by Morgan [3,13], who showed that an iceberg profile (characterised by an above-average vigour score and below-average scores for tension, depression, anger, fatigue, and confusion) was predictive of successful performance. Subsequent studies have identified other distinct mood profiles among athletes, such as the Everest profile [4] (characterised by near-maximum scores for vigour and near-zero scores for tension, depression, anger, fatigue, and confusion), which—like the iceberg profile—has been linked with successful performance. Conversely, the inverse iceberg profile (characterised by a below-average vigour score and above-average scores for tension, depression, anger, fatigue, and confusion), has been associated with suboptimal performance and a heightened risk of psychopathology [14].

The BRUMS and the associated norms were developed on and for use by English-speaking respondents, which creates challenges for sport psychology practitioners who work in other language contexts. To ensure the effective application of mood profiling across cultures and countries, it is essential to translate the BRUMS to capture cultural and linguistic nuances. To do this, comprehensive translation and validation processes are required to extend the cross-cultural generalizability of the BRUMS. This has most recently been applied to a validation of the Lithuanian-language version of the Brunel Mood Scale (BRUMS-LTU) [15], with the BRUMS previously being translated and cross-validated in Afrikaans [16], Bangla [17], Brazilian Portuguese [18], Chinese [19], Czech [20], French [21], Hungarian [22], Italian [22,23], Japanese [24], Persian [25], Serbian [26], Spanish [27], and Turkish [28] contexts.

In a Malaysian context, two previous studies have tested Malay translations of the BRUMS [29,30], although both have limitations. For example, the Hashim et al. study [29] failed to provide details of the translation procedure, and the sample consisted of only adolescent athletes from one geographical location, the majority of whom competed in the sport of taekwondo. This raises questions about the generalizability of study results to other age groups (e.g., older athletes in Malaysia), other regions of the country, and athletes from other sports (e.g., field hockey, soccer). The Lane et al. study [30] was methodologically stronger having implemented a rigorous method to generate Malay mood descriptors. Additionally, the sample was larger and more diverse in comparison to the Hashim et al. study. The respondents were athletes taken from across Malaysia who together participated in more than 30 different sports. However, the sample included a high proportion of adolescent athletes, again raising questions about the generalizability of results to other age groups. Additionally, ethnicity was not considered in either study. As Malaysia is an ethnically diverse country, it is not clear if findings are representative of this diversity. Given these concerns, the utility and efficacy of the existing Malay translations of the BRUMS remains questionable. As highlighted by McGannon et al. [31], and Ryba et al. [32], cultural awareness and cultural competence are acknowledged as key elements of effective practice and delivery of sport psychology to address the requirements of participants from culturally diverse nations. The multicultural diversification underlying the Malaysian nation, and more specifically in the elite sports setting, provides a strong imperative to conduct further cross-cultural research in the Malaysian context.

Therefore, the primary purpose of our study was to validate a Malay translation of the BRUMS, referred to as the Malaysian Mood Scale (MASMS; See Appendix A). The psychometric properties of the MASMS were evaluated against the original measurement model of the BRUMS [11,12]. It was hypothesised that the MASMS subscale scores would highly correlate with concurrent measures of similar constructs (i.e., convergent validity) and show minimal correlation with concurrent measures of dissimilar constructs (i.e., divergent validity) [33]. It was also hypothesised that negatively valanced MASMS scales would correlate with concurrent measures of depression, anxiety, and stress [23]. The secondary purpose of our study, based on previous evidence of the influence of demographic variables on mood responses [34,35], was to test for differences in mood scores between athletes and non-athletes, males and females, and younger and older participants.

## 2. Materials and Methods

### 2.1. Participants

A total of 4923 Malay-speaking participants were involved in the study. The sample was socio-demographically heterogenous, with similar representation of males (54.97%; *n* = 2706) and females (45.03%; *n* = 2217), and a range of age groups, education levels, and states of origin (see Table 1). The ethnic distribution of participants was 46.50% Malay (*n* = 2289), 32.70% Chinese (*n* = 1608), 13.10% Indian (*n* = 645), with 7.70% selecting the “Other” ethnicity category (*n* = 381). The ethnic distribution of our sample approximated the distribution for Malaysia as a whole [36]. In sum, 52% (*n* = 2559) of respondents participated competitively in sport at international level (*n* = 856) or state level (*n* = 1703).

### 2.2. Measures

#### 2.2.1. Brunel Mood Scale (BRUMS)

The BRUMS is a 24-item scale made up of basic mood descriptors with a standard response time frame of “How do you feel right now?” Participants rate their responses on a 5-point Likert scale of 0 = Not at all, 1 = A little, 2 = Moderately, 3 = Quite a bit, and 4 = Extremely. The measure has six subscales (i.e., tension, depression, anger, vigour, fatigue, and confusion) with each containing four mood descriptors. The completion time for the BRUMS is approximately two minutes. Total subscale scores may range from zero to 16. Subscales are comprised of the following items:

Anger: annoyed, bitter, angry, and bad-tempered (i.e., items 7, 11, 19, 22).
Confusion: confused, mixed up, muddled, and uncertain (i.e., items 3, 9, 17, 24).
Depression: depressed, downhearted, unhappy, and miserable (i.e., items 5, 6, 12, 16).
Fatigue: worn out, exhausted, sleepy, and tired (i.e., items 4, 8, 10, 21).
Tension: panicky, anxious, worried, and nervous (i.e., items 1, 13, 14, 18).
Vigour: lively, energetic, active, and alert (i.e., items 2, 15, 20, 23).

Developed by Terry et al. [11,12], the BRUMS is one of the few variations of the original POMS that has undergone rigorous validity testing. Each of the six subscales have been validated via multisample confirmatory factor analysis (CFA) using four different samples: adult students (*n* = 656), adult athletes (*n* = 1984), young athletes (*n* = 676), and schoolchildren (*n* = 596; [11,12]). Comprehensive tables of normative data are available for each of the abovementioned four populations. The BRUMS has also demonstrated high internal consistency, with Cronbach coefficient alphas ranging from 0.74 to 0.90 for each subscale [11,12]. Test–retest reliability coefficients ranging from 0.26 to 0.53 over a one-week period have been reported, which is appropriate for a measure of transient psychological states [11,12].

#### 2.2.2. Depression Anxiety Stress Scale-21

The Malay-validated version [37] of the Depression Anxiety Stress Scale 21 (DASS-21) [38], which consists of 21 items rated on a 4-point Likert scale, was administered concurrently to a subsample of participants. High scores indicate high levels of depression, anxiety, and stress. The DASS-21 was chosen as a concurrent measure in the present study because the instrument has also been administered in previous validations studies of translated BRUMS versions, such as the Italian Mood Scale (ITAMS) [23].

### 2.3. Translation of the Brunel Mood Scale into Malay

To develop the MASMS, a group of bilingual (i.e., Malay and English) experts with sport and/or social psychology backgrounds used a translation–back-translation methodology [39], similar to that used in the development of the ITAMS [23]. Firstly, three highly proficient multilingual experts independently translated the BRUMS into the Malay language. With an aim to validate cultural representation and linguistic relatability, discrepancies between translations were discussed and reconciled to reach consensus. Following this, three different linguistic experts independently performed a back-translation of the agreed-upon scale from Malay into English [40]. Of note, all six experts were certified under the Malaysian Translation Association (MTA) and were experienced social science translators [41]. Next, a comparison was made between the original and back-translation versions of the BRUMS to ensure that all translated units accurately defined the initial intent of the source language [42,43]. This step in the process was completed by two psychology professionals who were proficient in both Malay and English. One of the original developers of the BRUMS, who is also the fourth author of the present study, provided guidance on operational, semantic, item, and conceptual equivalences during the finalisation of the translation.

Next, the methodology used by Zhang et al. [19] in the development of the Chinese version of the Brunel Mood Scale (BRUMS-C) was applied to ensure comprehensibility of items and instructions of the newly translated MASMS. Using convenience sampling, feedback was sought from 60 individuals (30 males, 30 females) from sport and general populations, aged 13–61 (*M* = 31.49 years, *SD* = 10.50). Minor textual and syntactic modifications were implemented based on the results of this field test. Proofreading was conducted by the first author to ensure that the titles, introduction, instructions for participants and the test administrator, mood items, scoring responses, and scoring instructions were accurate representations of the source-language questionnaire (BRUMS).

### 2.4. Alternative Word Lists of the Malaysian Mood Scale

To acknowledge the importance of item comprehension and to account for the language proficiency of individuals, a culturally appropriate alternative word list [44] was formulated (see Appendix B). The list was provided to minimise misunderstanding of the translated mood descriptors.

### 2.5. Procedure

The research protocol was approved by the Human Research Ethics Committee at the University of Southern Queensland in accordance with the Australian Code for the Responsible Conduct of Research [H14REA057]. Participants were recruited from sporting and general populations using snowball sampling over a 2-year period from November 2018 to February 2020. They were presented with details of the research purpose and informed consent was provided by all the participants prior to data collection. Participation was voluntary and participants were free to withdraw at any time. The alternative word lists of the MASMS were also presented to participants who required linguistic support in better understanding scale items. To assess test–retest reliability and concurrent validity, a sample of 302 participants completed the MASMS a second time along with the Malay version of the DASS-21 [37]. Demographic data (i.e., age, sex, ethnicity, state of origin, level of education, sport participation, types of sport, level of participation) were also collected in both instances.

### 2.6. Data Analysis

Statistical analyses were conducted using IBM SPSS (IBM Corp, Armonk, NY, USA) and AMOS Statistics (IBM Corp, Chicago, IL, USA) for Windows, version 27.0 [45,46]. The factorial validity of the MASMS was assessed using CFA, by testing how well the hypothesised measurement model of the BRUMS [11] fitted the sample covariance matrix of the MASMS. Adequate internal consistency and goodness-of-fit measures are essential to corroborate with the factor structure to ensure the cultural adaptation of the MASMS is evaluated thoroughly. The concurrent validity of the MASMS was evaluated using the DASS-21 [37] as an external reference. Based on previous findings of Terry et al. [11,12], positive relationships were hypothesised between the negative mood scores of the MASMS (tension, depression, anger, fatigue, and confusion) and the depression, anxiety, and stress subscales of the DASS-21. Negative relationships between the vigour scale of the MASMS and the depression, anxiety, and stress subscales of the DASS-21 were anticipated. Preliminary tables of normative data for the MASMS were also developed. To produce normative data tables for use in Malaysian contexts, raw scores on each MASMS subscale were converted to T-scores, using the formula: T = 50 + (10 × z) [47]. Finally, multivariate analysis of variance (MANOVA) was used to test for differences in mood responses when participants were grouped by sport participation (athletes vs. non-athletes), sex (males vs. females), and age group (younger [≤27 years] vs. older [28+]) participants.

## 3. Results

Significant univariate abnormality was identified in some negatively valenced MASMS subscales (i.e., tension, depression, anger, confusion). This was consistent with mood subscale distributions in previous BRUMS datasets [47,48], as negative mood dimensions typically show a larger proportion of scores at the lower end, and fewer scores at the upper end [11,12]. Abnormality has also been reported in past BRUMS validation studies [23,49], with adequate model fit being obtained without data transformation. Further, in line with the recommendation of Nevill and Lane [50] that self-report measures should not be transformed with measurement scales at the interval level, no data transformations occurred prior to the analysis. A total of 103 significant multivariate outliers (*p* < 0.001) were identified via the Mahalanobis distance test. However, no examples of response bias in the form of straight-line, acquiescent, or extreme responding were detected [51,52]. Subsequently, all outliers were retained, and a final sample of 4923 cases were included in the analyses.

### 3.1. Confirmatory Factor Analysis

**Figure 1:**
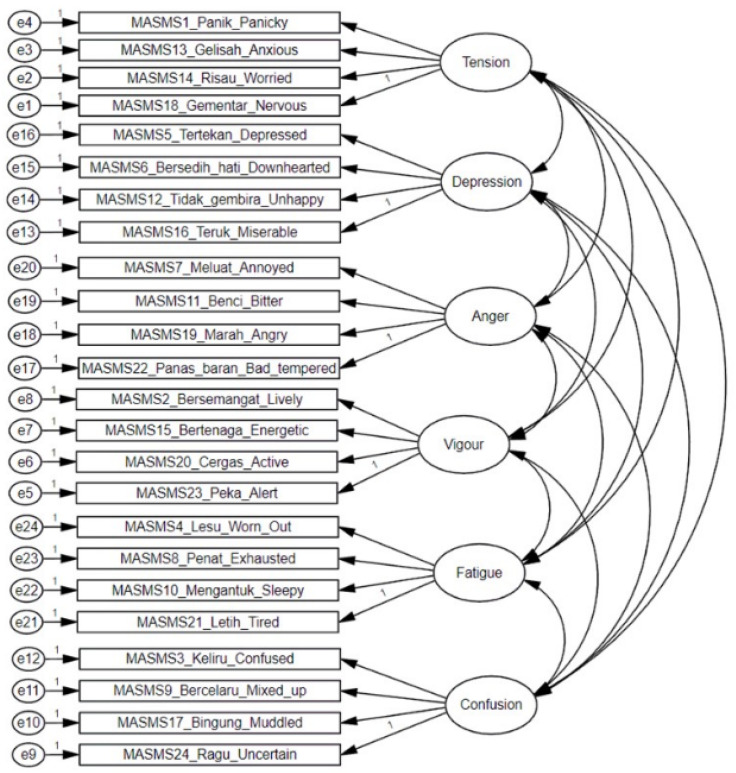
Six-factor model of the Malaysian Mood Scale.

Results of the CFA to evaluate the adequacy of the MASMS measurement model are shown in Table 2. A single-factor model (i.e., one factor of 24 items) was identified to be a poor fit (CFI = 0.603, TLI = 0.673, RMSEA = 0.158), whereas a six-factor model (i.e., six factors of four items each) showed acceptable fit (CFI = 0.949, TLI = 0.941, RMSEA = 0.067). Akaike’s information criterion statistic (AIC) [53] of the six-factor model (AIC = 5562.56) strengthened its superiority over the single-factor model (AIC = 13,259.55), and hence all subsequent analyses of the MASMS were based on the six-factor measurement model (as presented in Figure 1).

Modification indices showed that the measurement model would be improved significantly if the error terms for two confusion terms (confused and mixed up), two fatigue terms (sleepy and tired) and two depression terms (depressed and downhearted) were allowed to covary. All these covariance pathways were consistent with the findings of previous validation studies [11,12,23,49]. The modified six-factor measurement model of the MASMS showed improvement in fit indices (CFI = 0.950, TLI = 0.940, RMSEA = 0.056, 90% CI (0.055, 0.058). CFA was also conducted on subsamples to test the measurement model independently among sex, age group, and sport participation.

Multisample analysis was conducted to test measurement invariance on several subsamples: (a) athlete vs. non-athlete, (b) male vs. female, and (c) younger (≤27 years) vs. older (28+ years) participants. The rationale of grouping participants into younger vs. older using 27 years as the cut-off point was to generate approximately equal-sized subsamples for subsequent analyses (see Table 1). As indicated in Table 2, fit indices for the subsample analyses showed good fit of the measurement model to the data, thus supporting factorial invariance across sport participation, sex, and age.

The descriptive statistics, reliabilities and intercorrelations among the six MASMS subscales are presented in Table 3. All subscales with a negative orientation (i.e., tension, depression, anger, fatigue, confusion) were significantly intercorrelated and correlated inversely with the vigour scores. Cronbach alpha coefficients for all six subscales were above 0.84, exceeding the threshold of acceptability [54].

### 3.2. Generation of Norms

**Figure A1:**
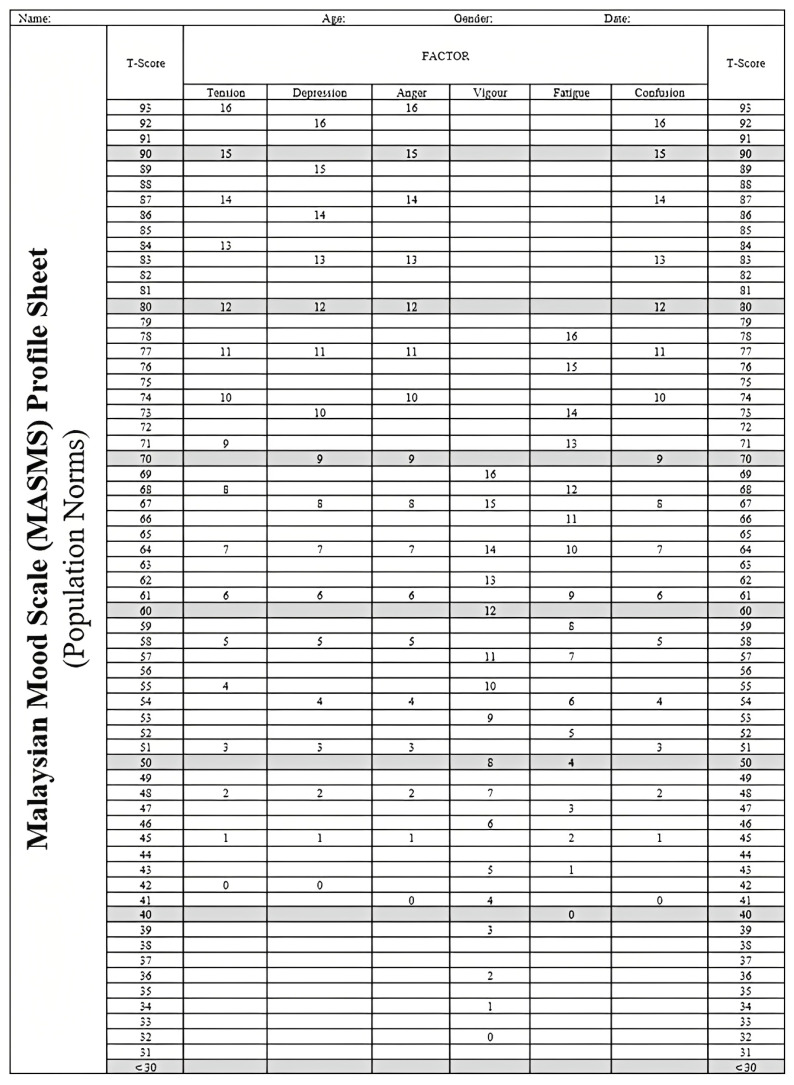
MASMS profile sheet (population norms).

**Figure A2:**
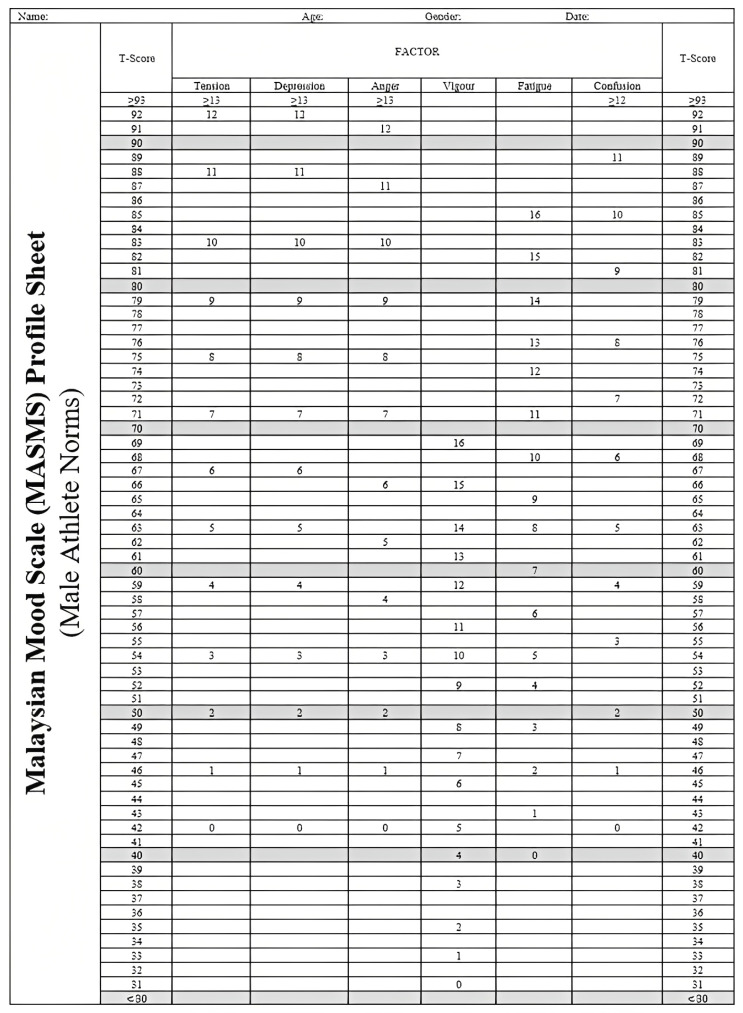
MASMS profile sheet (male athlete norms).

**Figure A3:**
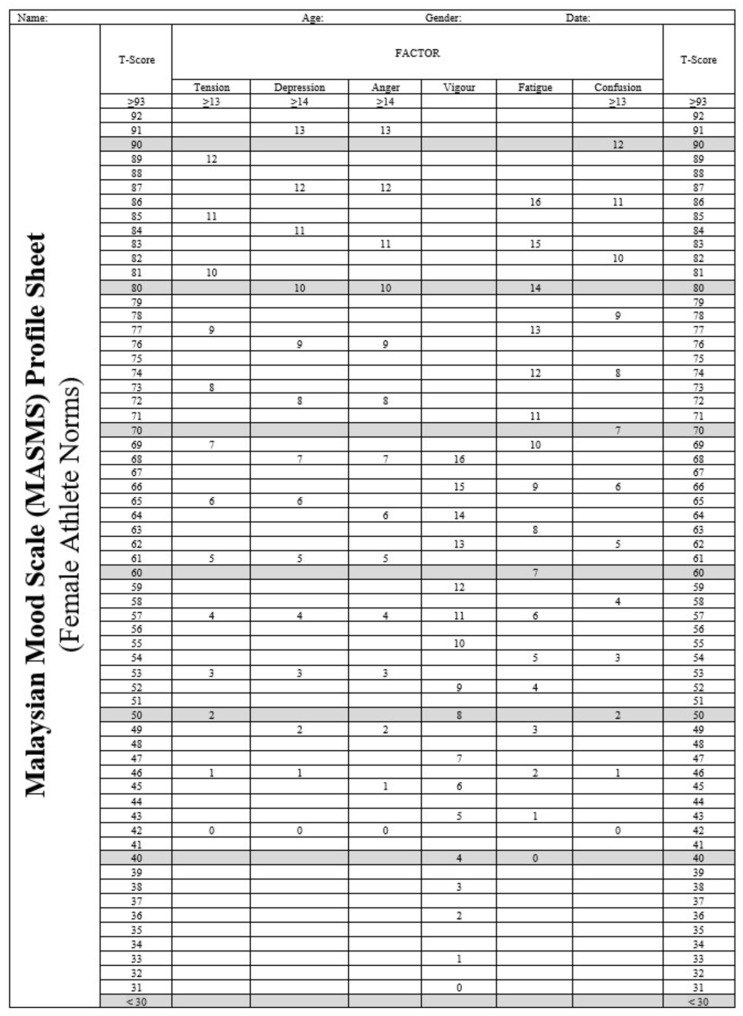
MASMS profile sheet (female athlete norms).

**Figure A4:**
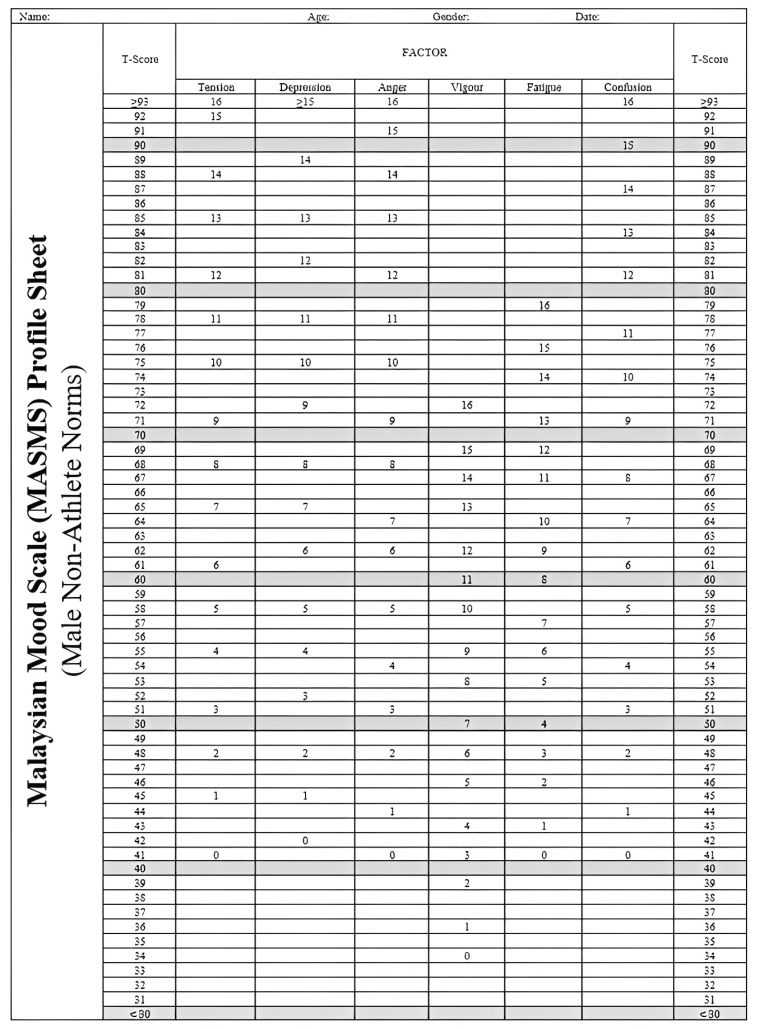
MASMS profile sheet (male non-athlete norms).

**Figure A5:**
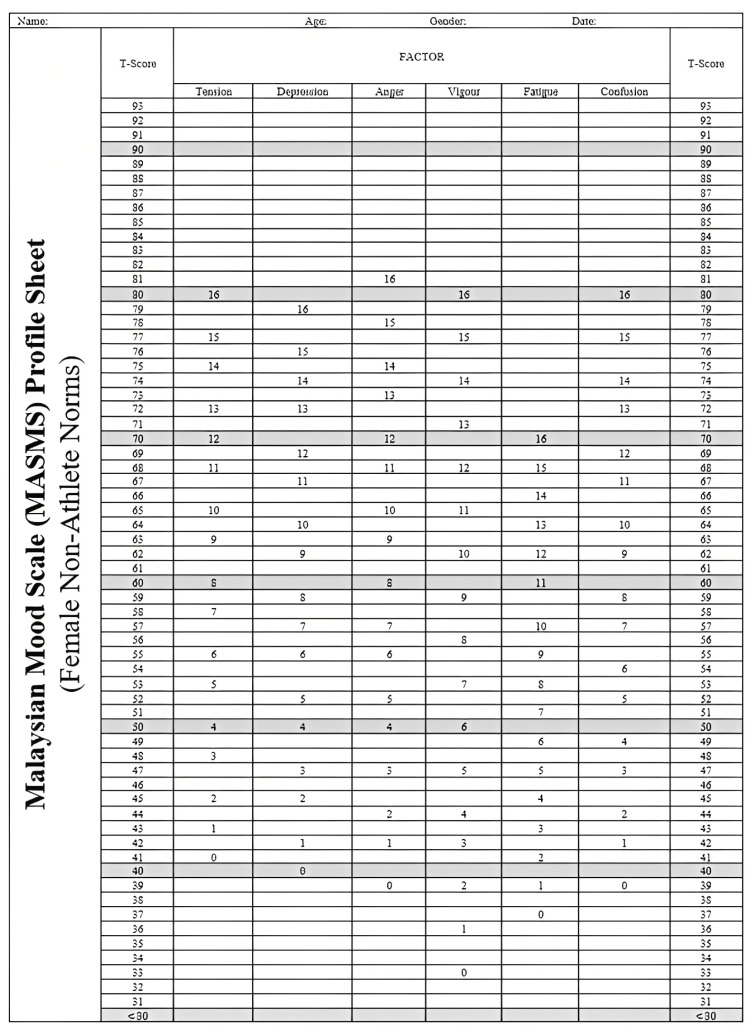
MASMS profile sheet (female non-athlete norms).

Preliminary tables of normative data were also generated (see Table 4,Table 5,Table 6,Table 7 and Table 8). Consistent with the study by Terry and Parsons-Smith [35], the generation of group-specific MASMS norms was restricted in this study to sex and sport participation. The norms reflected differences in raw scores both within and across groups. For example, among male athletes, a T-score of 42 equates to a raw score of 0 for tension, depression, anger, and confusion, but a raw score of 5 for vigour (see Table 5). Correspondingly, a T-score of 94 equates to a raw score of 12 for confusion among male athletes, but a raw score of 13 among female athletes (see Table 5 and Table 6). To assist practitioners and researchers in applying the MASMS in Malaysia and to facilitate the interpretation of mood scores, mood profile sheets include the specific norms in a format that enables the profile for an individual or team to be plotted diagrammatically (see Figure A1, Figure A2, Figure A3, Figure A4 and Figure A5 in Appendix C).

### 3.3. Concurrent Validity and Test–Retest Reliability

To explore the concurrent validity of the measure, relationships among the six subscales of the MASMS (i.e., tension, depression, anger, vigour, fatigue, and confusion) and the three subscales of the DASS-21 (i.e., depression, anxiety, and stress), bivariate correlations were conducted on a sample of 302 participants who also completed the Malay version of the DASS-21. The observed relationships were consistent with theoretical predictions (see Table 9). Large effects (i.e., correlations above 0.50) [55] were evident between the tension, depression, anger, and confusion subscales of the MASMS, and all three subscales of the DASS-21, thereby demonstrating convergent validity. The MASMS fatigue subscale showed a medium effect (0.30–0.50) with each of the DASS-21 subscales. Conversely, the MASMS vigour scale showed medium-to-large inverse relationships with DASS-21 subscales, thereby demonstrating divergent validity.

To assess the test–retest reliability of the MASMS, a sample of 302 participants also completed the MASMS for a second time, with an intervening period of 1–2 weeks. It was identified that the test–retest coefficients for the six subscales of the MASMS ranged from 0.48 to 0.62, which were almost identical to those reported previously [12] and deemed to be appropriate for a measure of transient psychological states.

### 3.4. Between-Group Comparisons

MANOVA was used to test for differences in mood responses when participants were grouped by sport participation, sex, and age group (see Table 10). Significant differences in mood responses were identified for sport participation (Hotelling’s T = 0.169, F [6, 4910] = 138.50, *p* < 0.001, $\eta_{p}^{2}\;$ = 0.145), accounting for 14.5% of the variance. Athletes reported higher scores for vigour and lower scores for anger, confusion, depression, fatigue, and tension than non-athletes. Males reported more positive moods than females (Hotelling’s T = 0.031, F [6, 4910] = 25.37, *p* < 0.001, $\eta_{p}^{2}\;$ = 0.030), with higher vigour scores coupled with lower anger, confusion, depression, fatigue, and tension scores, accounting for 3.0% of the variance. For age group (Hotelling’s T = 0.023, F [6, 4910] = 18.78, *p* < 0.001, $\eta_{p}^{2}\;$ = 0.022), younger participants (≤27 years) reported higher scores for vigour and fatigue, and lower scores for confusion and tension than older participants (28+ years), accounting for 2.2% of the variance.

## 4. Discussion

Our primary purpose was to validate a Malay language version of the BRUMS. The factorial validity, internal consistency, concurrent validity, and test–retest validity of the MASMS were evaluated in a Malay-speaking sample, which consisted of athlete and non-athlete participants. The six-factor measurement model was supported, with fit indices providing evidence of adequate model fit (see Table 2). Multisample CFA analyses supported factorial invariance across subsamples grouped by sport participation, sex, and age group.

Factor intercorrelations were in line with theoretical predictions (see Table 3). The negative orientation subscales of tension, depression, anger, fatigue, and confusion were all significantly intercorrelated and inversely correlated with vigour scores. The convergent and divergent validity of the MASMS was supported via relationships with depression, anxiety, and stress as measured by the Malay version of the DASS-21. Negatively-valenced MASMS scales correlated with DASS-21 subscales, demonstrating convergent validity, and the MASMS vigour scale correlated negatively with DASS-21 subscales, demonstrating divergent validity. The test–retest reliability of the MASMS was also supported.

Development of the MASMS reinforces the importance of conducting research with culturally appropriate measures [31,32] and offers a range of applications for researchers and applied practitioners who work in a Malaysian context. From a research perspective, the MASMS provides a measure of mood with comprehensible terminology and adequate attention to cultural nuances, thereby creating an impetus for mood-related research with standardised measures within the ethnic and cultural diversity of the Malaysian setting. The validated MASMS provides increased opportunity to conduct multicultural research in Malaysia, notably testing and possibly updating Lane and Terry’s conceptual model of mood–performance relationships [2], Morgan’s mental health model [56], and replicating research on the predictive effectiveness of mood assessments on performance in sports such as aikido [57], field hockey [58], karate [59], swimming [60], and triathlon [61]. The MASMS could also be used to investigate the prevalence of the previously identified six mood profile clusters, namely, the iceberg, inverse iceberg, inverse Everest, surface, submerged, and shark-fin profiles [35,48,62], among the Malaysian population. Another future research direction would be to investigate how the six mood profiles [35,48,62] affect performance among Malaysian athletes. The brevity of the MASMS promotes mood assessment in research environments with limited time availability for data collection, specifically prior to competition or during intervals of sporting events, and helps to support the initiation of relevant individualised mood management strategies.

There has been increased attention on mental health and well-being in a sporting context, especially for athletes competing at the elite level [63,64,65,66,67,68,69]. For example, a qualitative study looking at the mental health of Malaysian elite athletes [69] argued that experiencing stressful physical and psychological demands during training and competition placed athletes at risk of developing adverse moods that negatively affected their mental health and psychological status. Further, a call to develop a more comprehensive framework to foster athletes’ mental health and well-being [70] suggests a need to better identify and intervene early to prevent mental health issues. Therefore, with the potential of implementing mood profiling as an indicator of psychopathology risk [71], the MASMS could be an effective mental health screening assessment to identify and monitor mood states of athletes in Malaysian sports. This may go some way towards achieving sustainable athlete psychological well-being. In clinical domains, future studies may include the MASMS to assess prevalence of mental health issues [71,72], as a measure for medical screening protocols [73], and to monitor cardiopulmonary and metabolic rehabilitation patients [74] in the Malaysian healthcare system.

For the applied practitioner, multifaceted applications of mood profiling in the sport domain (refer [4] for a review) may also benefit sporting athletes and teams in Malaysia. Terry [4] suggested that regular mood profiling can function as an effective mechanism for sport psychology practitioners in monitoring athlete mindset. It can serve as a catalyst for discussion in one-to-one sessions, as a systematic way to monitor optimal training load, assess reactions to acclimatisation, as an indicator of general wellness, during the injury rehabilitation process, and for performance prediction among elite performers. Further, the importance of understanding idiosyncratic relationships between mood and performance has also been emphasised [4]. Replicating the approach used with the BRUMS [75], a user-friendly manual of the MASMS should be generated to provide a reference for practitioners and researchers for the application of the MASMS in Malaysia. The tables of normative data (see Table 4, Table 5, Table 6, Table 7 and Table 8) generated as a part of the present study will assist in the interpretation of MASMS raw scores. To generate graphical representation and interpretation of individual mood profiles, the standardised scores can be plotted on the relevant profile sheet (see Figure A1, Figure A2, Figure A3, Figure A4 and Figure A5 in Appendix C). There is scope to introduce evidence-based mood-regulation techniques where appropriate.

With increased emphasis placed on the importance of monitoring athlete mental health status and personal well-being [63,64,65,66,67,68,69], the MASMS could be used as an efficient self-report measure of mood for monitoring training load responses to reduce risk of overtraining and burnout [76,77], especially given the rigorous demands of training and competition. Further, a recent meta-analysis by Trabelsi et al. [78] reported significant mood deterioration, specifically in the form of increased fatigue and decreased vigour, among athletes who continued to train and compete whilst also observing the food and fluid restrictions of the Muslim holy month of Ramadan. Given that many of Malaysia’s elite athletes are Muslims who similarly observe these restrictions, there may be benefits associated with increased monitoring of their mood during the annual Ramadan period.

Regarding our secondary purpose, significant differences in mood responses were identified for sport participation, sex, and age. Athletes in our sample reported more positive moods than non-athletes, with significant differences on all six subscales, which is consistent with previous findings [23,49]. Positive mood can be promoted through engagement in aerobic exercise [79] and long-term physical activity [80], both of which would be typical behaviours for athletes. Overall, being physically active has well-established mood-enhancing effects [81,82,83,84,85], thereby alleviating manifestations of negative mood [86].

Variation in mood scores between males and females was also identified in our study. This is consistent with Iranian [25], Italian [23], South African [16], Serbian [26], Singaporean [87], and Spanish [27] studies, wherein females reported more negative moods than males, with lower scores for vigour, and higher scores for tension, depression, anger, fatigue, and confusion. This result is consistent with the findings of the Malaysian National Health and Morbidity Survey (NHMS) 2019 [88], wherein females reported a higher prevalence of mental health issues than males. On a global scale, it has been reported that females are nearly twice as likely to experience mental disorders as males [89], although this may be at least partially explained by the greater willingness of females to seek professional assistance for mental health issues [90]. Among the explanations for sex differences in mood responses is the potential of mood disturbance linked to endocrine changes associated with females’ reproductive life cycle (e.g., menstruation, pregnancy, menopause) [91,92] and the experience of mood disorders due to societal challenges (e.g., workforce inequality, sex discrimination) [93] that are more prevalent among females. It has also been identified that males are less likely than females to engage in rumination [94] and are less likely to report negative feelings (e.g., nervous, overwhelmed, depressed) [95] than females, although there is evidence that males tend to conceal symptoms of mental ill health which may lead to under-reporting and under-diagnosis of negative moods [96].

In relation to age, our results are inconsistent with some previous studies. Older participants in the current study reported higher scores than younger participants for tension and confusion and lower scores for vigour and fatigue, whereas the reverse has been found in English-speaking and Singaporean samples [34,49]. However, our results are consistent with Malaysian age-group findings in the NHMS report [88], in which older adults (30–74 years old) had a higher prevalence (15.3%) of mental health issues than younger adults (15–29 years old; 9.1%). Elderly Malaysians tend to have less formal education and lower fitness levels than their younger counterparts [88], characteristics that may increase mental health risk. In our sample, participants aged 50–75 years had the highest level of “no formal education” or “primary education only,” and generally did not participate in sporting activities. Unfortunately, health literacy in Malaysia is curtailed for older and less educated groups [88]; thus, older citizens may be oblivious to the knowledge that involvement in sport and exercise can help protect against mental ill-health [79,80,81,82,83,84,85,86]. Use of the MASMS as a mental health screening tool among all Malaysians, but particularly those in the older age groups, may prove beneficial in identifying “at risk” individuals at an early stage, which is noted by the World Health Organization (WHO) as an important intervention in promoting healthy ageing [97].

Some limitations of our study should be acknowledged. Firstly, despite gathering one of the largest samples among BRUMS translation studies, all data were collected prior to the COVID-19 pandemic. The lived experience of COVID pandemic restrictions has caused widespread mood deterioration [98], which may restrict the relevance of the tables of normative data to the Malaysian population at the current time. It is recommended that further research using the MASMS be conducted to assess whether refinement of norms is required. This would also enable researchers to track the impact of COVID on mental health in Malaysia. Given that the COVID-19 pandemic is not yet over, it would also be fruitful to conduct studies to explore the impact of pandemic challenges (e.g., physical distancing, lockdown, economic fallout, travel restriction) on mood disturbance, which may be beneficial in identifying effective coping strategies to reduce any negative impact the pandemic may have on mental well-being. A second limitation relates to the age of the participants, as no mood-profiling data were obtained from individuals under 17 years of age. According to the demographic statistics reported by DOSM [36], approximately 23% of the total population of Malaysia is <15 years old. Therefore, assessing the mood of the participants in that age group, as done in previous studies [11,19], would enhance the generalisability of the MASMS to a wider age range.

Finally, additional investigation of the antecedents, correlates, and behavioural consequences of mood responses among athletes and non-athletes in Malaysia is suggested. The interaction between socio-demographic factors and health status (e.g., availability of social support services, place of residence, level of household income, marital status, dietary habits, physical condition, amount of physical activity conducted) may provide further insights into the mood profiles among the Malaysian population across the age distributions. Extending the investigation of the MASMS among targeted groups of participants beyond the world of sport and exercise (e.g., youth, seniors) across various contexts, including academia, health professions, and the military, would be informative in expanding the range of mood-profiling applications in a Malaysian context.

## 5. Conclusions

Overall, our findings support the factorial, convergent, and divergent validity of the MASMS and its internal consistency. The tables of normative scores and mood-profile sheets can be used to guide the interpretation of mood scores and to monitor mental health status among Malaysian athletes and the general population. As a result, we conclude that the MASMS is a well-validated version of the BRUMS for use in Malay-language contexts. Finally, our findings showed significant differences in mood scores between athletes and non-athletes, males and females, and younger and older participants. Hence, we conclude that such demographic differences should be considered when interpreting mood scores.

## Figures and Tables

**Table 1:** Demographic distribution of the sample (*n* = 4923).

Source	Group	*n*	%
Sex	Male	2706	55.0%
	Female	2217	45.0%
Ethnic Distribution	Malay	2289	46.5%
	Chinese	1608	32.7%
	Indian	645	13.1%
	Other	381	7.7%
Age Group	≤27 years	2609	53.0%
	28+ years	2314	47.0%
Participation	Athlete	2559	52.0%
	Non-athlete	2364	48.0%
Education	Non-formal	75	1.5%
	Primary	153	3.1%
	Secondary	2807	57.0%
	Undergraduate	1760	35.8%
	Postgraduate	128	2.6%
State of Origin	Perlis	237	4.8%
	Kedah	342	6.9%
	Penang	385	7.8%
	Perak	350	7.1%
	Kuala Lumpur	423	8.6%
	Selangor	510	10.4%
	Negeri Sembilan	322	6.5%
	Melaka	314	6.4%
	Johor	384	7.8%
	Pahang	345	7.0%
	Kelantan	343	7.0%
	Terrengganu	290	5.9%
	Sabah	317	6.4%
	Sarawak	361	7.3%

**Table 2:** Model testing of the MASMS (*n* = 4923).

Group	*x* ^2^	*df*	CFI	TLI	RMSEA	90% CI
Full sample one-factor	12,593 *	252	0.603	0.673	0.158	[0.156, 0.159]
Full sample six-factor	5430 *	234	0.949	0.941	0.067	[0.066, 0.069]
Full sample six-factor modified	5329 *	232	0.950	0.940	0.056	[0.055, 0.058]
Multi-sample 1 (Sport Participation)	6132 *	468	0.947	0.938	0.049	[0.048, 0.050]
Multi-sample 2 (Sex)	6028 *	468	0.946	0.936	0.049	[0.048, 0.050]
Multi-sample 3 (Age Group)	6355 *	468	0.944	0.934	0.051	[0.049, 0.052]

Note: CFI = comparative fix index, TLI = Tucker–Lewis index, RMSEA = root mean square error of approximation, CI = confidence interval. Full sample (*N* = 4923), multisample 1: athlete (*n* = 2559) vs. non-athlete (*n* = 2364); multi-sample 2: male (*n* = 2706) vs. female (*n* = 2217); multi-sample 3: age ≤ 27 years (*n* = 2609) vs. age 28+ years (*n* = 2314). * *p* < 0.01. The six-factor modified model allowed covariance between the error terms for two confusion terms (confused and mixed up), two Fatigue terms (sleepy and tired) and two depression terms (depressed and downhearted).

**Table 3:** Descriptives, reliabilities and intercorrelations among MASMS subscales (*n* = 4923).

Subscale	*M*	SD	Range	T-Score	α	2	3	4	5	6
1 Anger	2.68	3.08	0–15	41–90	0.87	0.91 *	0.90 *	0.54 *	0.90 *	−0.09 *
2 Confusion	2.65	3.10	0–15	41–90	0.88		0.92 *	0.54 *	0.90 *	−0.12 *
3 Depression	2.60	3.15	0–15	42–89	0.89			0.53 *	0.89 *	−0.12 *
4 Fatigue	4.15	4.24	0–15	40–76	0.92				0.47 *	−0.31 *
5 Tension	2.57	3.10	0–16	42–93	0.85					−0.07 *
6 Vigour	7.85	4.32	0–16	32–69	0.92					

Note: * *p* < 0.01.

**Table 4:** MASMS normative scores for the whole sample (*n* = 4923).

Raw Score	T-Score					
	Tension	Depression	Anger	Vigour	Fatigue	Confusion
0	42	42	41	32	40	41
1	45	45	45	34	43	45
2	48	48	48	36	45	48
3	51	51	51	39	47	51
4	55	54	54	41	50	54
5	58	58	58	43	52	58
6	61	61	61	46	54	61
7	64	64	64	48	57	64
8	68	67	67	50	59	67
9	71	70	70	53	61	70
10	74	73	74	55	64	74
11	77	77	77	57	66	77
12	80	80	80	60	68	80
13	84	83	83	62	71	83
14	87	86	87	64	73	87
15	90	89	90	67	76	90
16	93	92	93	69	78	92

**Table 5:** MASMS normative scores for the male athlete sample (*n* = 1388).

Raw Score	T-Score					
	Tension	Depression	Anger	Vigour	Fatigue	Confusion
0	42	42	42	31	40	42
1	46	46	46	33	43	46
2	50	50	50	35	46	50
3	54	54	54	38	49	55
4	59	59	58	40	52	59
5	63	63	62	42	54	63
6	67	67	66	45	57	68
7	71	71	71	47	60	72
8	75	75	75	49	63	76
9	79	79	79	52	65	81
10	83	83	83	54	68	85
11	88	88	87	56	71	89
12	92	92	91	59	74	94
13	96	96	95	61	76	98
14	100	100	99	63	79	102
15	104	104	103	66	82	105
16	107	107	106	69	85	109

**Table 6:** MASMS normative scores for the female athlete sample (*n* = 1171).

Raw Score	T-Score					
	Tension	Depression	Anger	Vigour	Fatigue	Confusion
0	42	42	42	31	40	42
1	46	46	45	33	43	46
2	50	49	49	36	46	50
3	53	53	53	38	49	54
4	57	57	57	40	52	58
5	61	61	61	43	54	62
6	65	65	64	45	57	66
7	69	68	68	47	60	70
8	73	72	72	50	63	74
9	77	76	76	52	66	78
10	81	80	80	55	69	82
11	85	84	83	57	71	86
12	89	87	87	59	74	90
13	93	91	91	62	77	94
14	97	95	95	64	80	98
15	99	98	98	66	83	101
16	102	101	101	68	86	104

**Table 7:** MASMS normative scores for the male non-athlete sample (*n* = 1318).

Raw Score	T-Score					
	Tension	Depression	Anger	Vigour	Fatigue	Confusion
0	41	42	41	34	41	41
1	45	45	44	36	43	44
2	48	48	48	39	46	48
3	51	52	51	41	48	51
4	55	55	54	43	50	54
5	58	58	58	46	53	58
6	61	62	62	48	55	61
7	65	65	64	50	57	64
8	68	68	68	53	60	67
9	71	72	71	55	62	71
10	75	75	75	58	64	74
11	78	78	78	60	67	77
12	81	82	81	62	69	81
13	85	85	85	65	71	84
14	88	89	88	67	74	87
15	92	93	91	69	76	90
16	95	96	94	72	79	93

**Table 8:** MASMS normative scores for the female non-athlete sample (*n* = 1046).

Raw Score	T-Score					
	Tension	Depression	Anger	Vigour	Fatigue	Confusion
0	41	40	39	33	37	39
1	43	42	42	36	39	42
2	45	45	44	39	41	44
3	48	47	47	42	43	47
4	50	50	50	44	45	49
5	53	52	52	47	47	52
6	55	55	55	50	49	54
7	58	57	57	53	51	57
8	60	59	60	56	53	59
9	63	62	63	59	55	62
10	65	64	65	62	57	64
11	68	67	68	65	60	67
12	70	69	70	68	62	69
13	72	72	73	71	64	72
14	75	74	75	74	66	74
15	77	76	78	77	68	77
16	80	79	81	80	70	80

**Table 9:** Descriptive statistics and reliabilities for DASS-21 subscales and two-tailed correlations with MASMS subscales (*n* = 302).

	DASS—Depression	DASS—Stress	DASS—Anxiety
*M*	6.92	7.39	5.86
SD	4.73	4.82	4.58
Range	0–21	0–21	0–21
**α**	0.91	0.89	0.85
Anger	0.61 *	0.52 *	0.55 *
Confusion	0.62 *	0.59 *	0.60 *
Depression	0.78 *	0.69 *	0.65 *
Fatigue	0.42 *	0.39 *	0.44 *
Tension	0.52 *	0.57 *	0.65 *
Vigour	−0.58 *	−0.46 *	−0.42 *

Note: * *p* < 0.001.

**Table 10:** MANOVAs of MASMS subscale scores by sport participation, sex, and age group.

Sport Participation (*n* = 4923)						
Subscale	Athlete (*n* = 2559)		Non-Athlete (*n* = 2364)		*F*	$\eta_{p}^{2}$
	*M*	*SD*	*M*	*SD*		
Anger	2.08	2.53	3.33	3.48	209.97 *	0.04
Confusion	1.99	2.40	3.36	3.59	249.59 *	0.05
Depression	2.03	2.52	3.22	3.62	184.00 *	0.04
Fatigue	3.45	3.57	4.91	4.75	150.36 *	0.03
Tension	2.03	2.47	3.14	3.57	162.06 *	0.03
Vigour	9.18	4.26	6.41	3.91	563.61 *	0.10
**Sex (*n* = 4923)**						
Subscale	**Male (*n* = 2706)**		**Female (*n* = 2217)**		*F*	$\eta_{p}^{2}$
	*M*	*SD*	*M*	*SD*		
Anger	2.33	2.73	3.11	3.41	80.39 *	0.02
Confusion	2.28	2.72	3.10	3.46	85.20 *	0.02
Depression	2.20	2.72	3.10	3.54	100.85 *	0.02
Fatigue	3.64	3.95	4.78	4.50	90.07 *	0.02
Tension	2.26	2.72	2.94	3.46	59.81 *	0.01
Vigour	8.08	4.41	7.57	4.19	17.36 *	0.00
**Age Group (*n* = 4923)**						
Subscale	**≤27 years (*n* = 2609)**		**28+ years (*n* = 2314)**		*F*	$\eta_{p}^{2}$
	*M*	*SD*	*M*	*SD*		
Anger	2.65	2.98	2.72	3.20	0.81	0.00
Confusion	2.55	2.93	2.76	3.29	5.72 **	0.00
Depression	2.56	3.02	2.65	3.29	1.18	0.00
Fatigue	4.47	4.28	3.79	4.18	31.01 *	0.01
Tension	2.47	2.98	2.67	3.22	4.80 **	0.00
Vigour	8.13	4.36	7.54	4.26	22.30 *	0.01

Note: * *p* < 0.001, ** *p* < 0.05.

## Data Availability

The data supporting the conclusions of this article will be made available by the corresponding author upon reasonable request.

## Appendix A

Arahan: Berikut merupakan senarai penyataan yang menggambarkan perasaan anda. Sila baca setiap penyataan secara teliti. Kemudian sila tandakan (X) di dalam kotak yang menggambarkan perasaan anda ketika ini. Sila pastikan anda menjawab setiap soalan. (Instructions: Below is a list of words that describe feelings. Please read each one carefully. Then mark the box (X) that best describes how you feel right now. Make sure you answer every question).

**Table A1:** Malaysian Mood Scale (MASMS).

No.	Perasaan (Mood Item)	Tiada Langsung (Not at All)	Ada Sedikit (A Little)	Sederhana (Moderately)	Ada (Quite a Bit)	Sangat Banyak (Extremely)
1.	Panik (Panicky)	[ ]	[ ]	[ ]	[ ]	[ ]
2.	Bersemangat (Lively)	[ ]	[ ]	[ ]	[ ]	[ ]
3.	Keliru (Confused)	[ ]	[ ]	[ ]	[ ]	[ ]
4.	Lesu (Worn out)	[ ]	[ ]	[ ]	[ ]	[ ]
5.	Tertekan (Depressed)	[ ]	[ ]	[ ]	[ ]	[ ]
6.	Bersedih Hati (Downhearted)	[ ]	[ ]	[ ]	[ ]	[ ]
7.	Meluat (Annoyed)	[ ]	[ ]	[ ]	[ ]	[ ]
8.	Penat (Exhausted)	[ ]	[ ]	[ ]	[ ]	[ ]
9.	Bercelaru (Mixed up)	[ ]	[ ]	[ ]	[ ]	[ ]
10.	Mengantuk (Sleepy)	[ ]	[ ]	[ ]	[ ]	[ ]
11.	Benci (Bitter)	[ ]	[ ]	[ ]	[ ]	[ ]
12.	Tidak Gembira (Unhappy)	[ ]	[ ]	[ ]	[ ]	[ ]
13.	Gelisah (Anxious)	[ ]	[ ]	[ ]	[ ]	[ ]
14.	Risau (Worried)	[ ]	[ ]	[ ]	[ ]	[ ]
15.	Bertenaga (Energetic)	[ ]	[ ]	[ ]	[ ]	[ ]
16.	Teruk (Miserable)	[ ]	[ ]	[ ]	[ ]	[ ]
17.	Bingung (Muddled)	[ ]	[ ]	[ ]	[ ]	[ ]
18.	Gementar (Nervous)	[ ]	[ ]	[ ]	[ ]	[ ]
19.	Marah (Angry)	[ ]	[ ]	[ ]	[ ]	[ ]
20.	Cergas (Active)	[ ]	[ ]	[ ]	[ ]	[ ]
21.	Letih (Tired)	[ ]	[ ]	[ ]	[ ]	[ ]
22.	Panas Baran (Bad-tempered)	[ ]	[ ]	[ ]	[ ]	[ ]
23.	Peka (Alert)	[ ]	[ ]	[ ]	[ ]	[ ]
24.	Ragu (Uncertain)	[ ]	[ ]	[ ]	[ ]	[ ]

## Appendix B

**Table A2:** Alternative Word List for the Malaysian Mood Scale.

Brunel Mood Scale Items	Malaysian Mood Scale Items	Alternative Word List
Panicky	Panik	Kelam-Kabut
Lively	Bersemangat	Keghairahan
Confused	Keliru	Kacau-Bilau
Worn Out	Lesu	Tidak Bermaya
Depressed	Tertekan	Murung
Downhearted	Bersedih Hati	Kecewa
Annoyed	Meluat	Bengang
Exhausted	Penat	Jerih-Perih
Mixed up	Bercelaru	Tidak Tentu Arah
Sleepy	Mengantuk	Berasa Hendak Tidur
Bitter	Benci	Tidak Suka
Unhappy	Tidak Gembira	Pilu
Anxious	Gelisah	Cemas
Worried	Risau	Bimbang
Energetic	Bertenaga	Berkuasa
Miserable	Teruk	Sengsara
Muddled	Bingung	Keliru
Nervous	Gementar	Gentar
Angry	Marah	Meradang
Active	Cergas	Aktif
Tired	Letih	Lelah
Bad Tempered	Panas Baran	Rengus
Alert	Peka	Tangkas
Uncertain	Ragu	Tidak Pasti
